# Cranial nerve monitoring in endoscopic endonasal surgery of skull base tumors (observing of 23 cases)

**DOI:** 10.1186/s41016-018-0146-3

**Published:** 2018-12-21

**Authors:** A. N. Shkarubo, I. V. Chernov, A. A. Ogurtsova, V. E. Chernov, O. V. Borisov, K. V. Koval, D. N. Andreev

**Affiliations:** 1 Department of Neurooncology, Federal State Autonomous Institution “N.N. Burdenko National Medical Research Center of Neurosurgery” of the Ministry of Health of the Russian Federation, Moscow, Russia; 2 Department of Neurophysiology, Federal State Autonomous Institution “N.N. Burdenko National Medical Research Center of Neurosurgery” of the Ministry of Health of the Russian Federation, Moscow, Russia; 3 Department of Neurosurgery, N.N. Burdenko Main Military Clinical Hospital of the Ministry of Defense of the Russian Federation, Moscow, Russia; 4 Laboratory of Molecular Human Genetics, Federal Research and Clinical Center of Physical-Chemical Medicine of Federal Medical Biological Agency, Moscow, Russia

**Keywords:** Electromyography, T-EMG, Intraoperative cranial nerve identification, Endoscopic endonasal approach, Skull base tumor surgery

## Abstract

**Background:**

Preservation of anatomic integrity and function of the cranial nerves during the removal of skull base tumors is one of the most challenging procedures in endoscopic endonasal surgery. It is possible to use intraoperative mapping and identification of the cranial nerves in order to facilitate their preservation.

The purpose of this study was to evaluate the effectiveness of intraoperative trigger electromyography in prevention of iatrogenic damage to the cranial nerves.

**Methods:**

Twenty three patients with various skull base tumors (chordomas, neuromas, pituitary adenomas, meningiomas, cholesteatomas) underwent mapping and identification of cranial nerves during tumor removal using the endoscopic endonasal approach in Department of Neurooncology of Federal State Autonomous Institution “N.N. Burdenko National Medical Research Center of Neurosurgery” of the Ministry of Health of the Russian Federation from 2013 to 2018. During the surgical interventions, mapping and identification of the cranial nerves were carried out using electromyography in triggered mode. The effectiveness of the method was evaluated based on a comparison with a control group (41 patients).

**Results:**

In the main group of patients, 44 nerves were examined during surgery using triggered electromyography. During the study, the III, V, VI, VII, and XII cranial nerves were identified intraoperatively. Postoperative cranial nerve deficiency was observed in 5 patients in the study group and in 13 patients in the control group. The average length of hospitalization was 9 days.

**Conclusion:**

We did not receive statistically significant data supporting the fact that intraoperative identification of cranial nerves using trigger electromyography reduces the incidence of postoperative complications in the form of cranial nerve deficits (*p* = 0.56), but the odds ratio (0.6) suggests a less frequent occurrence of complications in the study group.

Based on our experience, the trigger electromyography methodology appears quite promising and requires further research.

## Introduction

Over the last 30 years, the microsurgical transsphenoidal approach has been widely used for removal of skull base tumors [1–4]. With the development of endoscopic technologies, the endoscopic endonasal transsphenoidal approach has become the gold standard for surgical removal of central skull base tumors [5–9].

During any surgical intervention on the base of the skull, there exists an inherent potential risk of damage to neurovascular structures, such as the internal carotid artery, anterior cerebral arteries, and cranial nerves, which can lead to temporary or permanent neurologic deficit [10].

The frequency of iatrogenic cranial nerve injuries in skull base surgery using different methods of intraoperative identification ranges from 2% to 47% [11–14]. Without neurophysiological identification, the incidence of cranial nerve injury has been reported to range from 14% to 68% [12, 15].

Neurological complications in the form of functional deficiency of the cranial nerves can be predicted and prevented using intraoperative neurophysiological monitoring [10, 16–18].

For the identification of cranial nerves, two main techniques are usually used: free-run electromyography (f-EMG) and triggered electromyography (t-EMG). F-EMG has a lower sensitivity in detecting cranial nerve deficits during endoscopic endonasal surgery of skull base tumors, since EMG activity is only observed after mechanical or electrical (cautery) manipulations on the cranial nerves [18, 19].

With t-EMG, changes occur after electrical stimulation of the cranial nerves by a monopolar or bipolar electrode, which leads to the formation of a compound muscle action potential—CMAP [17, 20]. Thus, t-EMG allows to determine the location of a cranial nerve before coming into direct contact with it, and, therefore, reduces the risk of iatrogenic damage during all stages of tumor removal [21].

In this regard, intraoperative identification of cranial nerves during transnasal endoscopic surgery is highly preferred. The purpose of this study was to evaluate the effectiveness of t-EMG in preventing intraoperative iatrogenic damage to the cranial nerves.

## Materials and methods

The presented study includes 23 patients (16 women and 7 men) of an average age of 52.9 years (26–72 years old), who underwent surgical treatment at department of neurooncology of Federal State Autonomous Institution “N.N. Burdenko National Medical Research Center of Neurosurgery” of the Ministry of Health of the Russian Federation from 2013 to 2018. During all surgical interventions in the study group, mapping and neurophysiological identification of the cranial nerves in the triggered mode were carried out.

The criteria for the inclusion of patients in the study group were as follows: age from 18 to 75 years, intradural extension of the tumor of the base of the skull, extension of the tumor into the region of localization of the cranial nerves, and intraoperative neurophysiological identification of at least one cranial nerve.

The technique of intraoperative identification of the cranial nerves used by us was described in detail in a previously published paper [22]. Briefly, for identification, needle electrodes are inserted into the muscles innervated by the corresponding nerves. For the mapping and identification of cranial nerves, rhythmic electrostimulation using solitary pulses with a frequency of 4.7 Hz, and a stimulus duration of 0.1 ms is carried out. The current varies from 2 to 16 mA. The registration of muscle motor responses is carried out in triggered EMG mode with an analysis period of 20 ms and sensitivity of 50 μV/Div. As anesthetic support, total intravenous anesthesia (TIVA) technology is used. For tracheal intubation, a muscle relaxant (rocuronium 0.6 mg/kg) of moderate duration is used.

To evaluate the effectiveness of the method, the data was compared with that in a control group of 41 patients (23 men, 18 women) with an average age of 46.3 years (16–73 years old).

**Fig. 1:**
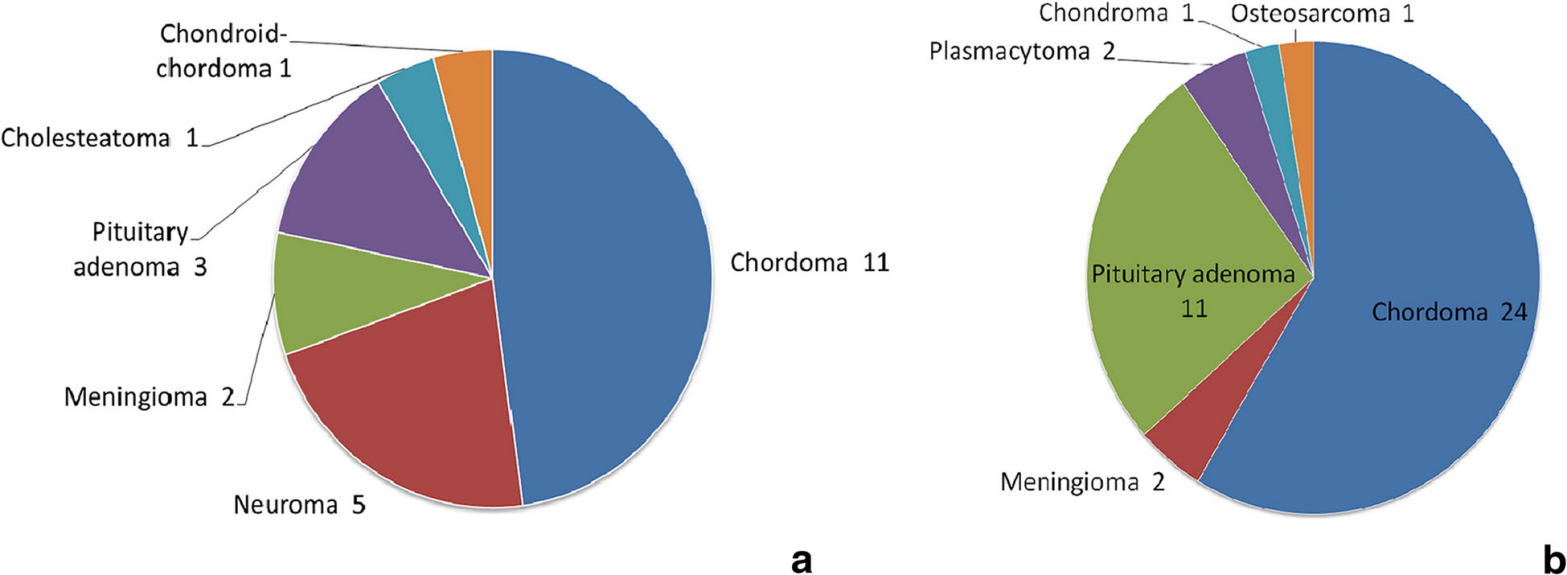
Patient distribution by diagnosis. **a** Study group (*n* = 23). **b** Control group (*n* = 41)

All patients were operated on by the same neurosurgeon at the department specializing on skull base surgery. The distribution of patients in the control and study groups according to diagnosis is presented in Fig. 1.

The neurologic status (including functional activity of the cranial nerves) of patients was evaluated before surgery, on the first day after surgery, and at follow-up examinations.

CT scan of the brain on the first day after surgery was performed in all cases.

For the control group, similar inclusion criteria were used, but intraoperative identification of the cranial nerves was not carried out.

The degree of tumor resection was evaluated according to the radicality scale proposed by Frank G. and Pasquini E. (2002):

1. Radical removal, when there are no signs of a tumor on contrast-enhanced CT and/or MRI images.
2. Subtotal removal, when the remaining portion of the tumor is less than 20% of the original tumor size.
3. Partial removal, when the remaining portion is less than 50% of the original tumor size.
4. Insufficient removal, when the remaining portion of the tumor is 50% or more of the original tumor size.

When evaluating the degree of tumor resection, CT and/or MRI data at the time of discharge were compared with the data of the control studies at 3 and 6 months after surgery.

Data on the nature of the surgery, depending on the location of the tumor, and on the initial neurologic status and postoperative complications are presented in Table 1.

**Table 1:** Cranial nerve monitoring and mapping statistics and corresponding current parameters

Patient	Diagnosis (histologic)	Localization	Symptoms before surgery	Approach	Tumor removal radicality	Cranial nerve identification	Symptom dynamics	
							At discharge	3 months after surgery
Patient 1. 63 years old	Trigeminal nerve neurinoma	Meckel’s cave	Paresis of CNs III, V on the right	Extended lateral (D)	Total	V and VII on the right	Paresis of CN III on right remained, *increased paresis of CN VI on right up to plegia*, CN V failure remained as well	Regression of CN VI right paresis
Patient 2.50 years old	Chordoma	Clivus area	Paresis of CNs III, VI on the right	Posterior extended transclival	Total	III bilaterally, VI on the left	Without dynamics	Partial regression of oculomotor disorders
Patient 3.49 years old	Trigeminal nerve neurinoma	Left cavernous sinus	Paresis of CNs III, IV, VI on the left	Extended lateral (L)	Total	VI and V on the left	Partial regression of oculomotor disorders	Further regression of oculomotor disorders
Patient 4.72 years old	Chordoma	Endo-supra-infra-latero (R, L)sellar	Chiasmatic syndrome, plegia of CN III on the left	Transsellar + extended bilateral	subtotal	VI bilaterally	Without dynamics	n/a
Patient 5.59 years old	Trigeminal nerve neurinoma	Right cavernous sinus	Hypoesthesia in projection of all 3 branches of CN V on right	Extended lateral (R)	Total	VI and V on the right	Partial regression of hypoesthesia	Without dynamics
Patient 6.66 years old	Chordoma	Clivus area	Left hemiparesis: arm—2, leg—3. Paresis of CNs V and VII on the left. Left hemi-hypoesthesia	Posterior extended transclival	Total	III bilaterally, VI on the right	Partial regression of left hemiparesis: arm—3, leg—4	Partial regression of left hemiparesis: arm—4, leg—4. Regression of VII left nerve paresis
Patient 7.26 years old	Chordoma	Clivus area and left cavernous sinus	Paresis of CNs V and VI on the left	Posterior extended transclival	Total	III on the left	Partial regression of VI left nerve paresis	Without dynamics
Patient 8.65 years old	Chordoma	Endo-supra-infrasellar	Headache	Posterior extended transclival + extended bilateral	Total	VI bilaterally	Regression of headache	Without dynamics
Patient 9.70 years old	Chordoma	Both cavernous sinuses	VIS OS = 0.01	Extended bilateral	Total	VI on the left	VIS OS = 0.03	VIS OS = 0.04
Patient 10.20 years old	Chordoma	Clivus area	Paresis of CN VI on the left	Posterior extended transclival	Total	VI bilaterally	Partial regression of VI left nerve paresis	Without dynamics
Patient 11.59 years old	Pituitary adenoma	Endo-supra-latero(R)sellar	Chiasmatic syndrome	Extended lateral (R)	Total	III on right, VI on the right	Without dynamics	Without dynamics
Patient 12.67 years old	Chordoma	Clivus area	Paresis of CN VI on the left	Posterior extended transclival	Total	V and VI on the left	Partial regression of VI left nerve paresis. Regression of headache	Without dynamics
Patient 13.31 years old	Chordoma	Clivus area	Paresis of CN VI on the left	Posterior extended transclival	Total	VI on the left	Regression of headache	Without dynamics
Patient 14.26 years old	Chordoma	Clivus area	Paresis of CN VI on the left	Posterior extended transclival	Total	VI bilaterally	Regression of VI left nerve paresis	Without dynamics
Patient 15.47 years old	Trigeminal nerve neurinoma	Right cavernous sinus	Paresis of CN V on the right	Extended lateral (R)	Total	VI on the right	Without dynamics	Without dynamics
Patient 16.65 years old	Trigeminal nerve neurinoma	Right cavernous sinus	Paresis of CN III on the right	Extended lateral (R)	Total	III, VI, V on the right	*Increased paresis of CN III on right*	Without dynamics
Patient 17.54 years old	Recurrent chordoma	Clivus area, left cavernous sinus	Paresis of CN VI on the left	Extended lateral (L)	Total	III, VI on the left	Partial regression of VI left nerve paresis	Regression of VI left nerve paresis
Patient 18.62 years old	Meningioma	Clivus area	Paresis of CN XII on the right	Posterior extended transclival	Subtotal	VI on the left	Regression of XII right nerve paresis	Without dynamics
Patient 19.33 years old	Cholesteatoma	Clival area	Paresis of CNs V, VII, VIII, IX, on the left	Posterior extended transclival	Subtotal	III, VI, VII, XII on the left	*Increased paresis of CN VI on left*	Without dynamics
Patient 20.72 years old	Recurrent pituitary adenoma	Endo-supra-latero(D)sellar	Headache	Transsellar + extended lateral (R)	Total	III on the right	Negative dynamics of visual functions	Without dynamics
Patient 21.53 years old	Meningioma	Petroclival	Headache	Posterior extended transclival	Subtotal	VI on the left	*Increased paresis of CN VI on left*	Regression of VI left nerve paresis
Patient 22.45 years old	Chondroid-chordoma	Clival area	Paresis of CNs VI, V	Posterior extended transclival	Subtotal	III, V on the left	*Increased paresis of VI nerve*	N/a
Patient 23.63 years old	Pituitary adenoma	Endo-supra-infra-latero(S)sellar	Chiasmatic syndrome	Transsellar + extended lateral (S)	Total	III, VI on the left	Regression of III, VI nerves paresis	N/a

Italics displays complications

To compare the groups, Fisher’s exact test and the Cochran-Armitage trend test were used. The significance threshold (α) for rejecting the null hypothesis was set in accordance with the generally accepted value of 0.05. When comparing the groups, odds ratios (OR) and 95% confidence intervals (CIs) were also calculated. Statistical analysis was carried out in the programming language R version 3.4.2 using Rstudio version 1.1.383.

## Results

From 2013 to 2018, 23 study group patients and 41 control group patients were operated on at our institute. All surgical interventions were performed using the endoscopic endonasal transsphenoidal approach.

**Fig. 2:**
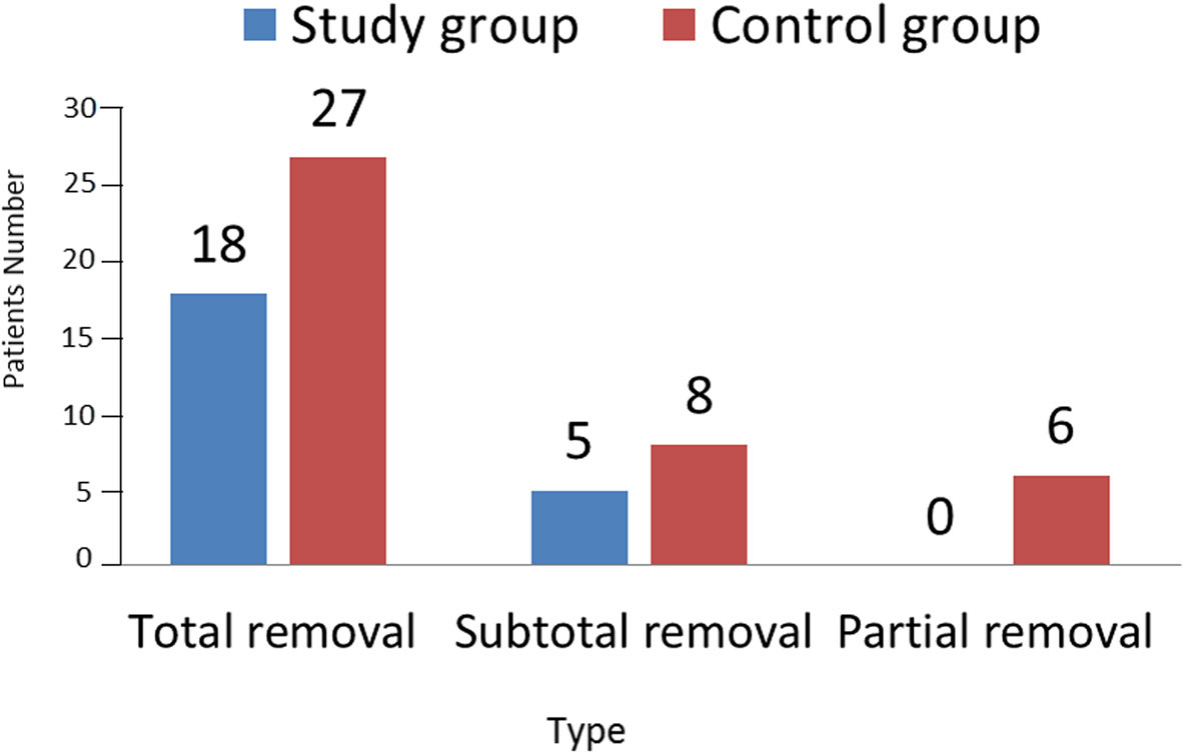
Results of surgical treatment based on control CT and/or MRI data

The results of surgical treatment were evaluated based on control CT and/or MRI data (Fig. 2). In the study group, 10 out of 11 chordomas were removed totally, and 1 subtotally, all neuromas (5) were removed totally, all meningiomas (2) were removed subtotally, all pituitary adenomas (3) were removed totally, and the cholesteatoma and chondroid-chordoma were removed subtotally. Thus, total removal of the tumor was achieved in 78% of the cases, and subtotal removal in 22% of the cases. In the control group, total tumor removal was achieved in 65.9% of the cases (27/41), subtotal removal in 19.5% (8/41), and partial removal in 14.6% (6/41) of the cases.

All patients in the study group underwent intraoperative identification of the cranial nerves (CN). The III, V, VI, VII, and XII cranial nerves were identified. The number of mapped cranial nerves is presented in Table 2.

**Table 2:** Cranial nerve monitoring and mapping statistics and corresponding current parameters

Cranial nerve	III	V	VI	VII	XII
Number of patients monitored	10	6	18	2	1
Number of mapped nerves	12	6	23	2	1
Average current	4–6 mA	4–10 mA	4–10 mA	4–10 mA	4–10 mA

In the study group, the incidence of complications in the form of cranial nerve function deficit amounted to 21.7% (5 patients). In one case, paresis of an intraoperatively unidentified nerve developed: CN VI was not identified intraoperatively in a patient with a trigeminal neuroma in the right cavernous sinus. The paresis was noted in the early postoperative period and regressed 3 months after the surgery. In three cases, paresis of intraoperatively identified cranial nerves developed.

**Fig. 3:**
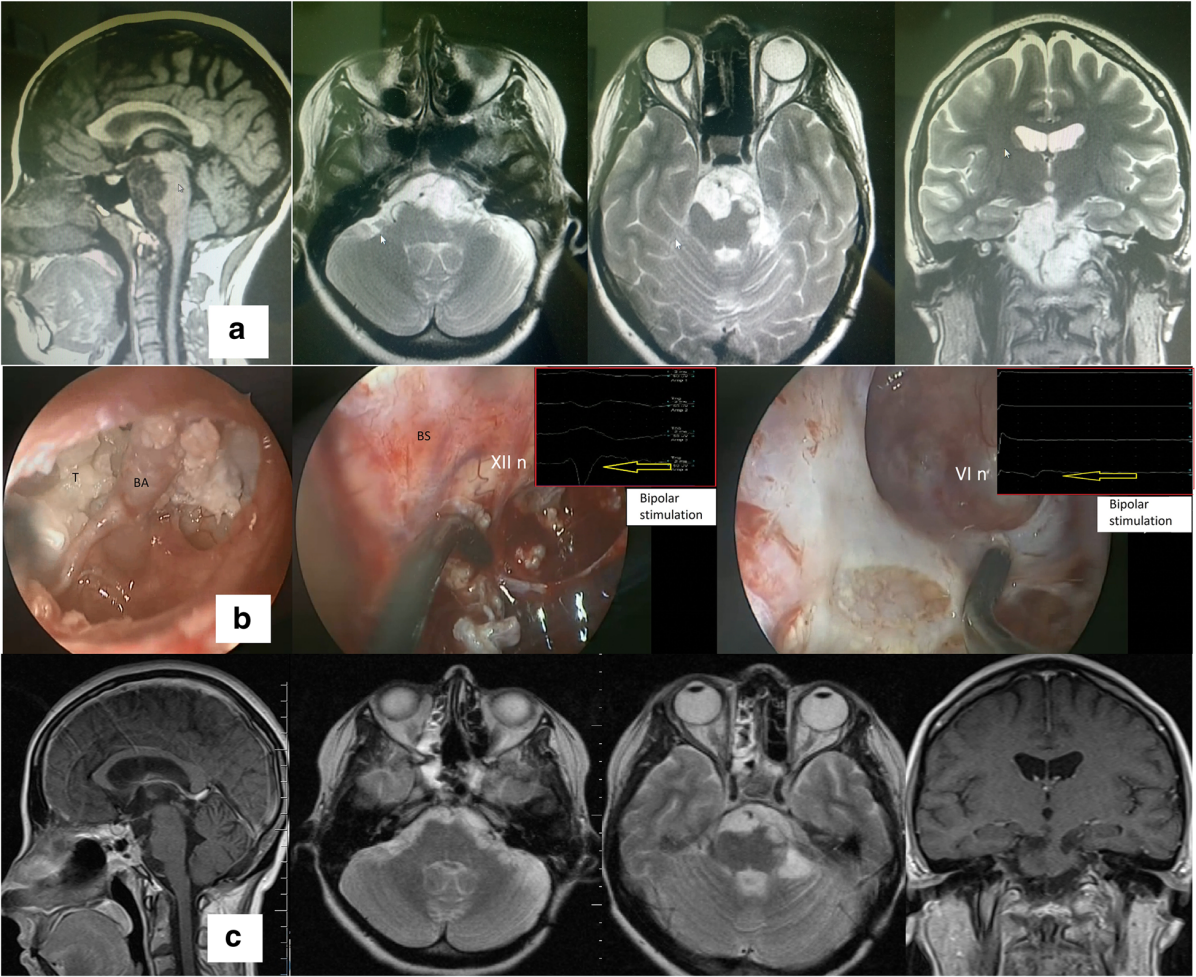
Clinical case. Patient no. 19 in Table 1. **a** MRI before surgery. Giant cholesteatoma in a 33 years old female patient (she was previously operated three times). **b** Intraoperative photograph. BA basilar artery, BS brain stem, T tumor. **c** MRI 4 months after surgery. Partial removal of the tumor

In the first case, paresis of CN III deteriorated in a patient with a neuroma of the right cavernous sinus, which did not regress within 3 months (before surgery the paresis was less pronounced). In the second case, paresis of CN VI developed in a patient with a giant cholesteatoma (Fig. 3) of the base of the skull, which also did not regress within 3 months (before the surgery there was no paresis). In the third case, paresis of CN VI developed in a patient with a giant meningioma of petroclival localization (there was no paresis before surgery). Detailed data on all clinical observations are presented in Table 1.

The incidence of complications in the form of cranial nerve function deficit in the control group was 31.7% (13 patients). In most of these cases (8/13), there was a new paresis or a deterioration of an existing paresis of the abducent nerve. In 6/13 cases, there was a new paresis or a deterioration of an existing paresis of the oculomotor nerve. One patient developed a paresis of the trochlear nerve (Table 3). Postoperative nerve injury was not related to brain tissue swelling or intraoperative area hemorrhage in any case (it was confirmed by CT on the first day after surgery).

**Table 3:** Postoperative complications in the study and control groups

	No complications	Paresis (paresis of identified nerves)
Study group	18	5 (3)
Control group	28	13 (−)

The average length of hospitalization was 9 days.

### Group comparison

The assessment of the statistical differences between the groups was carried out using an exact Fisher test (due to deviation from the theoretical chi-square distribution). When comparing the study and control groups with and without paresis of unidentified nerves, no significant differences were found (*p* values of 0.56 and 0.22, respectively). At the same time, the calculated odds ratios indicate a less frequent occurrence of complications in the study group: OR = 0.6 (CI 0.2–2.0) and 0.39 (CI 0.06–1.7) when comparing patients with and without paresis in unidentified nerves, respectively.

## Discussion

Because of the intimate proximity between the structures of the base of the skull and the neurovascular structures (internal carotid artery, anterior cerebral artery, and cranial nerves III to XII), even minimally invasive endoscopic endonasal surgical interventions are associated with the potential risk of their iatrogenic damage, which can lead to a decrease in the quality of life of the patients [23–25]. Due to this risk, monitoring of the functional integrity of cranial nerves during endoscopic endonasal surgery is of great importance [26].

For the purposes of this study, we used t-EMG for intraoperative mapping and identification of cranial nerves during endoscopic endonasal surgery of skull base tumors. In published studies, it has been shown that the use of t-EMG during surgical removal of various skull base tumors can reduce the risk of postoperative neurological complications associated with functional cranial nerve deficiency [19, 21].

The absence of M-responses when an impulse is applied to a CN may be a sign of complete damage of the nerve trunk. However, if M-responses can be obtained with increased current strength, partial nerve damage is likely. This phenomenon can serve as a predictor of postoperative deficiency [27]. In our study, postoperative deficits of the cranial nerves that were identified intraoperatively were observed in three cases. It must be noted, that the current strength was not increased for the identification of these nerves. The development of functional deficiency of the cranial nerves, which were identified intraoperatively, does not allow us to affirm the prognostic significance of the use of t-EMG at this stage of research.

Intraoperatively, we examined the functional integrity of cranial nerves III, V, VI, VII, and XII. Surgical treatment of tumors extending into the cavernous sinus, the upper orbital gap, and the petroclival region is associated with a significant risk of functional and structural damage to the extraocular cranial nerves [28–30]. Diplopia after intraoperative injury to the extraocular cranial nerves can have a serious impact on the quality of life of the patient, as loss of stereoscopic vision can be associated with a risk of visual field narrowing, secondary amblyopia, and even functional blindness [17, 20]. Functional deficit of CN IV leads to less significant defects in eye movement than deficit of cranial nerves III and VI [31]. Based on our observations (in the study group), the postoperative function of CN VI (three cases) deteriorated most often, which is in line with literature data [27, 32]. The extension of the endoscopic endonasal approach to the clival region also necessitates monitoring of the caudal group of cranial nerves [18, 33]. In one clinical observation (a giant skull base cholesteatoma), we were able to identify and map, as well as preserve the functional and anatomic integrity of CN XII.

In our study, intraoperative identification and mapping of cranial nerves did not reduce the radicality of the surgical interventions, but, in fact, allowed it to be extended (78% in the study group vs 65.9% in the control group).

We did not obtain a statistically significant confirmation that the intraoperative identification of cranial nerves using t-EMG reduces the incidence of postoperative complications in the form of cranial nerve deficit (*p* > 0.05), but the odds ratio (OR = 0.6) suggests a less frequent occurrence of complications in the study group. We speculate that the hypothesis centering on the notion of decreased postoperative complication rates due to the use of t-EMG can be validated through a study involving a bigger patient cohort.

## Conclusion

The results of our study did not provide a statistically significant confirmation that intraoperative identification of cranial nerves using t-EMG reduces the incidence of postoperative complications in the form of cranial nerve deficit (*p* = 0.56). However, the odds ratio (0.6) does indeed suggest a less frequent occurrence of complications in the group of patients, which underwent surgery with t-EMG support.

We consider the t-EMG methodology is promising, even though it unquestionably requires further research. More studies are needed with a selection of larger groups of patients to confirm or refute the importance of using the described technique in reducing the frequency of postoperative complications associated with cranial nerve deficiency.

## Abbreviations

CI: Confidence interval
CMAP: Compound muscle action potential
CN: Cranial nerve
EMG: Electromyography
f-EMG: Free-run electromyography
OR: Odds ratio
t-EMG: Triggered electromyography
TIVA: Total intravenous anesthesia

## References

[1] Bowers CA, Altay T, Couldwell WT (2011). Surgical decision-making strategies in tuberculum sellae meningioma resection. Neurosurg Focus.

[2] Arai H, Sato K, Okuda MM, Hishii M, Nakanishi H, Ishii H (2000). Transcranial transsphenoidal approach for tuberculum sellae meningiomas. Acta Neurochir.

[3] Couldwell WT, Weiss MH, Rabb C, Liu JK, Apfelbaum RI, Fukushima T (2004). Variations on the standard transsphenoidal approach to the sellar region, with emphasis on the extended approaches and parasellar approaches: surgical experience in 105 cases. Neurosurgery.

[4] de Divitiis E, Esposito F, Cappabianca P, Cavallo LM, de Divitiis O (2008). Tuberculum sellae meningiomas : high route or low route? A series of 51 consecutive cases. Neurosurgery.

[5] Mahmoud M, Nader R, Al- Mefty O (2010). Optic canal involvement in tuberculum sellae meningiomas : influence on approach, recurrence, and visual recovery. Neurosurgery.

[6] Cook SW, Smith Z, Kelly DF (2004). Endonasal transsphenoidal removal of tuberculum sellae meningiomas : technical note. Neurosurgery.

[7] Gardner PA, Kassam AB, Thomas A, Snyderman CH, Carrau RL, Mintz AH, Prevedello DM (2008). Endoscopic endonasal resection of anterior cranial base meningiomas. Neurosurgery.

[8] Liu JK, Christiano LD, Patel SK, Tubbs RS, Eloy JA (2011). Surgical nuances for removal of tuberculum sellae meningiomas with optic canal involvement using the endoscopic endonasal extended transsphenoidal transplanum transtuberculum approach. Neurosurg Focus.

[9] Jho HD, Ha HG (2004). Endoscopic endonasal skull base surgery: part 1—the midline anterior fossa skull base. Minim Invasive Neurosurg.

[10] Thirumala PD, Kassasm AB, Habeych M, Wichman K, Chang YF, Gardner P (2011). Somatosensory evoked potential monitoring during endoscopic endonasal approach to skull base surgery: analysis of observed changes. Neurosurgery.

[11] Maurer J, Pelster H, Ronald G, Amedee AM, Wolf J (1995). lntraoperative monitoring of motor cranial nerves in skull base surgery. Skull base surgery.

[12] Liang SQ, Liang EH, Chen BD, Chen L (2012). Intraoperative oculomotor nerve monitoring during skull base tumor surgery. Zhonghua Yi Xue Za Zhi.

[13] Parthasarathy D, Thirumala PD, Mohanraj SK, Habeych M, Wichman K, Chang YF, Gardner P, Snyderman C, Crammond DJ, Balzer J (2012). Value of free-run electromyographic monitoring of lower cranial nerves in endoscopic endonasal approach to skull base surgeries. J Neurol Surg B Skull Base.

[14] Sekhar LN, Møller AR (1986). Operative management of tumors involving the cavernous sinus. J Neurosurg.

[15] Wu B, Liu WD, Chen LY, Huang GF (2013). Application of far lateral craniocervical approach in the microsurgical treatment of the jugular foramen tumors. Zhonghua Wai Ke Za Zhi.

[16] Romstöck J, Strauss C, Fahlbusch R (2000). Continuous electromyography monitoring of motor cranial nerves during cerebellopontine angle surgery. J Neurosurg.

[17] Schlake HP, Goldbrunner RH, Milewski C, Krauss J, Trautner H, Behr R (2001). Intra-operative electromyographic monitoring of the lower cranial motor nerves (LCN IX-XII) in skull base surgery. Clin Neurol Neurosurg.

[18] Thirumala P, Lai D, Engh J, Habeych M, Crammond D, Balzer J (2013). Predictive value of somatosensory evoked potential monitoring during resection of intraparenchymal and intraventricular tumors using an endoscopic port. J Clin Neurol.

[19] Elangovan C, Singh SP, Gardner P, Snyderman C, Tyler- Kabara EC, Habeych M, Crammond D, Balzer J, Thirumala PD (2016). Intraoperative neurophysiological monitoring during endoscopic endonasal surgery for pediatric skull base tumors. J Neurosurg Pediatr.

[20] Sekiya T, Hatayama T, Iwabuchi T, Maeda S (1993). Intraoperative recordings of evoked extraocular muscle activities to monitor ocular motor nerve function. Neurosurgery.

[21] HARNER STEPHEN G., DAUBE JASPER R., EBERSOLD MICHAEL J., BEATTY CHARLES W. (1987). Improved Preservation of Facial Nerve Function With Use of Electrical Monitoring During Removal of Acoustic Neuromas. Mayo Clinic Proceedings.

[22] Shkarubo AN, Chernov IV, Ogurtsova AA, Moshchev DA, Lubnin AJ, Andreev DN, Koval KV (2017). Neurophysiological identification of cranial nerves during endoscopic Endonasal surgery of skull base tumors: pilot study technical report. World Neurosurg..

[23] Kassam AB, Gardner P, Snyderman C, Mintz A, Carrau R (2005). Expanded endonasal approach: fully endoscopic, completely transnasal approach to the middle third of the clivus , petrous bone, middle cranial fossa, and infratemporal fossa. Neurosurg Focus.

[24] Kassam A, Snyderman CH, Mintz A, Gardner P, Carrau RL (2005). Expanded endonasal approach: the rostrocaudal axis. Part I. Crista galli to the Sella turcica. Neurosurg Focus.

[25] Kassam A, Snyderman CH, Mintz A, Gardner P, Carrau RL (2005). Expanded endonasal approach: the rostrocaudal axis. Part II. Posterior clinoids to the foramen magnum. Neurosurg Focus.

[26] Singh H, Vogel RW, Lober RM (2016). Intraoperative neurophysiological monitoring for endoscopic endonasal approaches to the skull base: a technical guide. Scientifica.

[27] Kawaguchi M, Ohnishi H, Sakamoto T (1995). Intraoperative electrophysiologic monitoring of cranial motor nerves in skull base surgery. Surg Neurol.

[28] Eisner W, Fiegele T, Dolenc VV, Rogers L (2009). Neuromonitoring in central skull base surgery. Cavernous sinusDevolopments and future perspectives.

[29] Gao D, Fei Z, Jiang X, Zhang X, Liu W, Fu L, Li B, Liang J (2012). The microsurgical treatment of cranio -orbital tumors assisted by intraoperative electrophysiologic monitoring and neuronavigation. Clin Neurol Neurosurg.

[30] Lopez JR (2011). Neurophysiologic intraoperative monitoring of the oculomotor, trochlear, and abducens nerves. J Clin Neurophysiol.

[31] Møller AR (2011). Practical aspects of monitoring cranial motor nerves. Intraoperative Neurophysiological Monitoring.

[32] Sekiya T, Hatayama T, Iwabuchi T, Maeda S (1992). A ring electrode to record extraocular muscle activity during skull base surgery. Acta Neurochir.

[33] Morera VA, Fernandez-Miranda JC, Prevedello DM, Madhok R, Barges- Coll J, Gardner P, Carrau R, Snyderman CH, Rhoton AL, Kassam AB (2010). “Far-medial” expanded endonasal approach to the inferior third of the clivus : the transcondylar and transjugular tubercle approaches. Neurosurgery.

